# Caveolae as Potential Hijackable Gates in Cell Communication

**DOI:** 10.3389/fcell.2020.581732

**Published:** 2020-10-27

**Authors:** Maria Dudãu, Elena Codrici, Cristiana Tanase, Mihaela Gherghiceanu, Ana-Maria Enciu, Mihail E. Hinescu

**Affiliations:** ^1^Biochemistry-Proteomics Laboratory, Victor Babes National Institute of Pathology, Bucharest, Romania; ^2^Cell Biology and Histology Department, Carol Davila University of Medicine and Pharmacy, Bucharest, Romania; ^3^Clinical Biochemistry Department, Faculty of Medicine, Titu Maiorescu University, Bucharest, Romania

**Keywords:** caveolae, caveolins, cell communication, cell signaling, gatekeeper, hijack, oncogenic signal, clinical trials

## Abstract

Caveolae are membrane microdomains described in many cell types involved in endocytocis, transcytosis, cell signaling, mechanotransduction, and aging. They are found at the interface with the extracellular environment and are structured by caveolin and cavin proteins. Caveolae and caveolins mediate transduction of chemical messages via signaling pathways, as well as non-chemical messages, such as stretching or shear stress. Various pathogens or signals can hijack these gates, leading to infectious, oncogenic and even caveolin-related diseases named caveolinopathies. By contrast, preclinical and clinical research have fallen behind in their attempts to hijack caveolae and caveolins for therapeutic purposes. Caveolae involvement in human disease is not yet fully explored or understood and, of all their scaffold proteins, only caveolin-1 is being considered in clinical trials as a possible biomarker of disease. This review briefly summarizes current knowledge about caveolae cell signaling and raises the hypothesis whether these microdomains could serve as hijackable “gatekeepers” or “gateways” in cell communication. Furthermore, because cell signaling is one of the most dynamic domains in translating data from basic to clinical research, we pay special attention to translation of caveolae, caveolin, and cavin research into clinical practice.

## Introduction

The term “caveolae” is more than 60 years old and traces back to seminal electron microscopy studies conducted independently by G.E. Palade (Palade, 1953; Bruns and Palade, 1968) and E. Yamada of various tissues including endothelia (Yamada, 1955a,b; Smith and Ryan, 1972) and muscle (Merrillees, 1960; Zampighi et al., 1975; Sawada et al., 1978). For a long time, their function(s) remained elusive, until electron microscopy studies complemented by biochemical investigations led to *in vitro* functional studies and *in vivo* models. In 2001, Lisanti’s lab produced the first caveolin knockout (KO) mouse strain (Cav1^–/–^) (Razani et al., 2001). Further research revealed that caveolae and their scaffold proteins are involved in specific cellular processes, such as plasma microdomain organization and cell signaling (Boscher and Nabi, 2012)—in both normal cells (Sowa, 2012) or tumor cells (Hehlgans and Cordes, 2011)—or even in a specific kind of tumor (Parat and Riggins, 2012). Caveolae also are involved in cell migration and metastasis (Nunez-Wehinger et al., 2014), mechano-reception (Nassoy and Lamaze, 2012), and mechano-protection in certain tissues (Lo et al., 2015). Embryologic development is a less explored area in caveolin research (but for review, see Sohn et al., 2016).

Technological progress in cell imaging has improved our understanding of how biochemical components of caveolae assemble into a functional ultrastructural domain of the cell membrane. Knockout animal models have added more to a comprehensive picture of these microdomains. Tracking the literature describing all of these membrane microdomains means navigating a flood of publications on caveolae and their scaffold proteins, caveolins, and cavins. Of about 100 reviews published in the last 5 years, only a few offer an integrated view (Cheng and Nichols, 2016; Han et al., 2016; Busija et al., 2017; Lamaze et al., 2017; Bhogal et al., 2018; Filippini et al., 2018; Parton et al., 2018; Leo et al., 2020). An inventory of (un)resolved issues regarding caveolae is exquisitely summarized in a recent review unconventionally entitled “Caveolae: The FAQs” (Parton et al., 2020).

This review briefly summarizes the knowledge in caveolae cell signaling and assesses the status of these microdomains as both gatekeepers and gateways in cell communication. Furthermore, because cell signaling is one of the most dynamic domains in translating data from basic to clinical research, special attention will be paid to caveolae, caveolins, and cavins research translation into clinical practice.

## Ultrastructure of Caveolae, Related to Caveolins, and Cavins Expression

**FIGURE 1:**
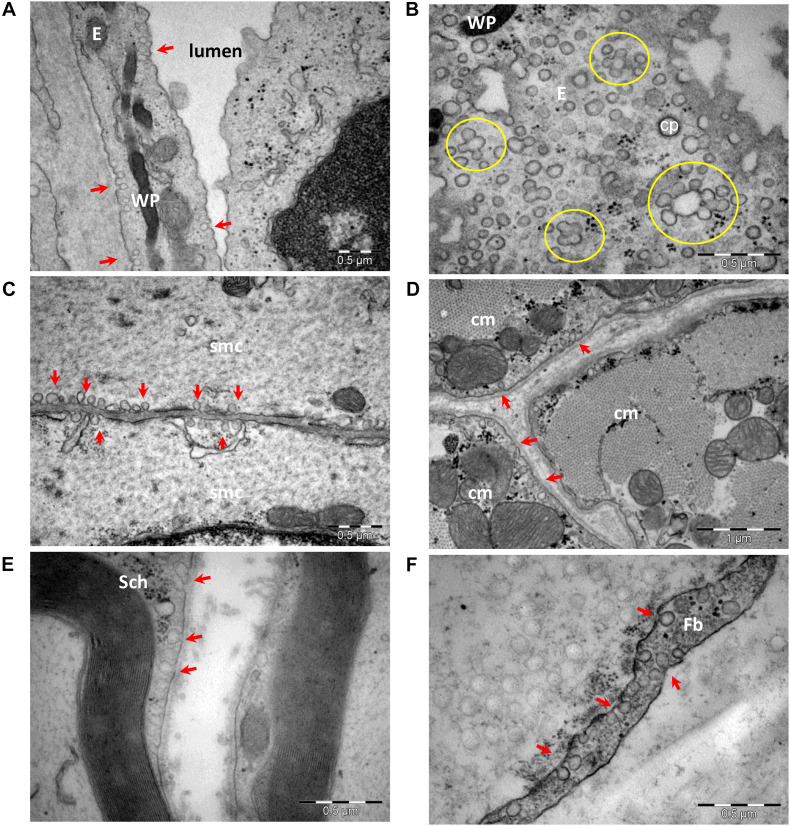
Transmission electron microscopy of cells with caveolae (arrows): endothelial cells **(A,B)**, smooth **(C)** and striated **(D)** muscle cells, Schwann cell **(E)**, and perineurial fibroblast **(F)**. Caveolae in a section parallel to an endothelial cell plasma membrane **(B)** shows numerous caveolae as single units or clustered forming rosettes (encircled). E, endothelial cell; cp, clathrin-coated pit; WP, Weibel–Palade bodies in endothelial cell; smc, smooth muscle cell; cm, cardiac muscle cell; Sch, Schwann cell; Fb, fibroblast (image collection, Department of Ultrastructural Pathology, “Victor Babes” Institute of Pathology, Bucharest).

Based on the first electron micrograph reports, caveolae were categorized and further evaluated as endocytotic vesicles (Gabella, 1978; Levin and Page, 1980; McGuire and Twietmeyer, 1983; Noguchi et al., 1987; Severs, 1988; Anderson, 1993). Organized as omega-shaped plasma membrane microdomains (segments of membrane with special lipid composition; Field, 2017), caveolae have been studied in experimental setups relying on cholesterol depletion (Liu and Pilch, 2008; Grundner and Zemljic Jokhadar, 2014). However, cholesterol is also enriched in other membrane microdomains, such as lipid rafts and chlatrin coated pits. Cholesterol depletion affects with variable degree a significant number of endocytotic pathways (reviewed in Thottacherry et al., 2019), thus generating results that lack specificity. Caveolae have been described in many cell types, including endothelial cells, smooth and striated muscle cells, interstitial cells of the heart (Gherghiceanu et al., 2009), adipose cells, fibroblasts, and Schwann cells of myelinated or unmyelinated peripheral nerve fibers (Figure 1). The number of caveolae varies in different cell types and has been reported to be up to 10,000/cell in endothelial cells (Couet et al., 1997c) and about 1,000,000/cell in adipocytes (Thorn et al., 2003). The number of caveolae also varies in smooth muscle cells of different tissues from Wistar rats, with 0.48 c/μm in the muscularis mucosa of stomach, 0.57 c/μm in the media of the aorta, 0.74 c/μm in the bladder, and 1.06 c/μm in the myometrium (Popescu et al., 2006). More so, the number of caveolae seems to be modified in pathological conditions, and a decreased number of caveolae in aortic smooth muscle cells has been associated with hypertension (Potje et al., 2019).

**FIGURE 2:**
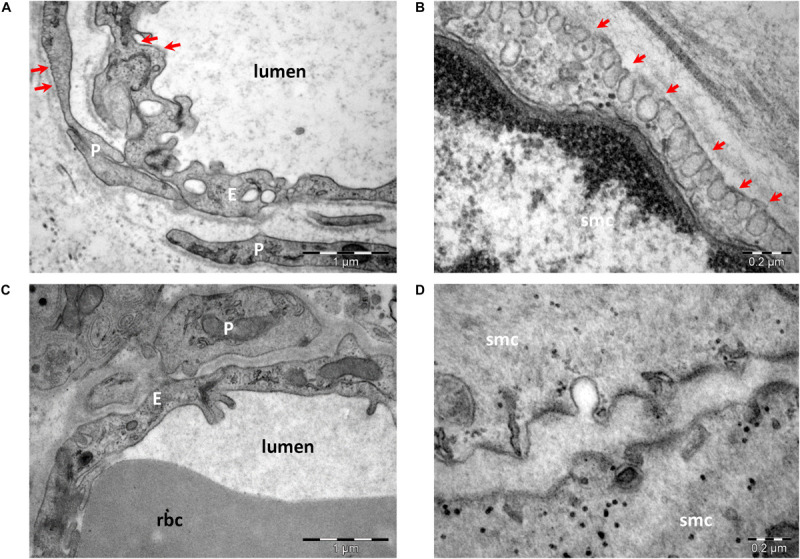
Transmission electron microscopy of endothelial cells **(A,C)** and smooth muscle cells **(B,D)** from wild-type **(A,B)** and Cav-1 KO **(C,D)** mice. Caveolae (arrows) are present in endothelial cells (E), pericytes (P), and smooth muscle cells (smc) in wild type **(A,B)**. Note the lack of caveolae in cells from Cav-1 KO mice **(C,D)** (image collection, Department of Ultrastructural Pathology, “Victor Babes” Institute of Pathology, Bucharest).

The protein scaffold composition of caveolae was resolved by mass spectrometry and cryoelectron tomography (Ludwig et al., 2013, 2016). The main scaffolding proteins are members of the caveolin family [caveolin (Cav)-1, -2, and -3], which associate as homo- or heterooligomers. The protein expression of caveolins differs in various tissues, as well as in their propensity to associate in heterooligomers. Caveolins have an even wider distribution, being detected even in cells that do not organize caveolae. Cav-1 is the main protein to form caveolae in non-muscle cells, alone or with Cav-2 (de Almeida, 2017), and its absence results in a lack of caveolae at least in endothelial and smooth muscle cells (Figure 2). Cav-2 KO mice show evidence of severe pulmonary dysfunction without disruption of caveolae (Razani et al., 2002). Cav-1/3 double-KO mice are viable but lack both muscle and non-muscle caveolae and develop a severe cardiomyopathic phenotype (Park et al., 2002). Cav-3 is the main scaffold protein of muscle cell membrane caveolae (Sohn et al., 2016). Mutations in this gene lead to skeletal muscle disease through multiple pathogenetic mechanisms, and Cav-3 deficiency has led to the recognition of a new category of pathologies labeled the caveolinopathies. These conditions include sarcolemmal membrane alterations, disorganization of the skeletal muscle T-tubule network, and disruption of distinct cell-signaling pathways. To date, 30 Cav-3 mutations have been identified in the human population. Cav-3 defects underlie four distinct skeletal muscle disease phenotypes: limb girdle muscular dystrophy, rippling muscle disease, distal myopathy, and hyperCKemia. In addition, one Cav-3 mutant has been described in a case of hypertrophic cardiomyopathy (Gazzerro et al., 2010).

**FIGURE 3:**
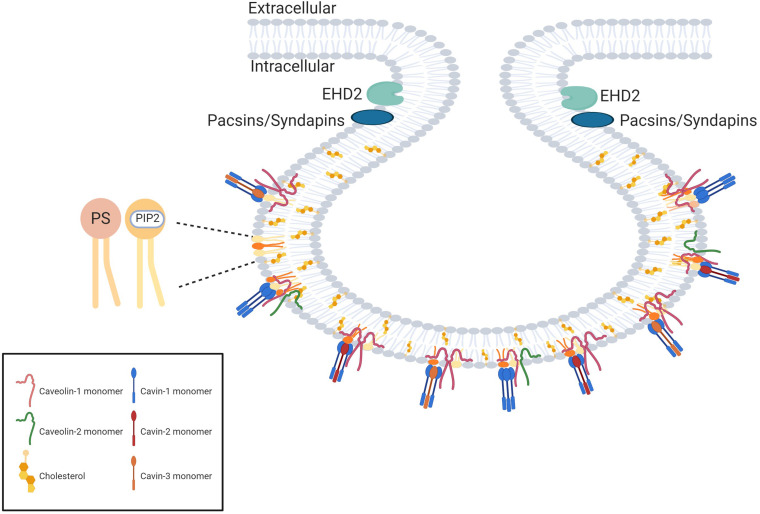
The protein scaffold of caveolae. The proteins forming the caveolae coat are named caveolins and cavins. The main proteins of the coat are caveolin-1 (which oligomerizes in homooligomers or heterooligomers with caveolin-2) and cavin-1 which forms homotrimers or heterotrimers with cavin-2 or cavin-3. The curvature of the neck of the caveolae is dependent on EH-domain containing protein 2 (EDH2) and pacsins. Created with BioRender.com.

In addition, cavin proteins (cavins 1–4) are recruited from cytosol to caveolae in the presence of caveolins and are required to stabilize the caveolar structure by multiple low-affinity interactions with caveolins and membrane lipids (Parton et al., 2018; Figure 3).

Similar to Cav-1, cavin-1 is required for caveolae formation, and KO of either leads to caveolae loss (Hill et al., 2008; Liu and Pilch, 2008). Cavin-2 deficiency in mice causes tissue-specific loss of caveolae, affecting lung endothelium and adipose tissue but not the endothelium in skeletal and cardiac tissue (Hansen et al., 2013). *In vitro*, cavin-2 induces membrane curvature in caveolae (Hansen et al., 2009), serves as part of a cholesterol sensor, and relocalizes cavin-1 from the cytosol to the plasma membrane (Breen et al., 2012). Cavin-3 does not play a major role in caveolae formation and composition (Liu et al., 2014a). Less is known about the pathogenic impact of cavin mutations in humans. Cavin-1 mutations cause secondary deficiency of caveolins, resulting in muscular dystrophy with generalized lipodystrophy (Hayashi et al., 2009). Also, muscle hypertrophy, muscle mounding, mild metabolic complications, and elevated serum creatine kinase levels have been observed in these patients (Dwianingsih et al., 2010; Rajab et al., 2010). Cavin-1 mutations also are associated with congenital general lipodystrophy type 4 (Shastry et al., 2010; Jelani et al., 2015; Patni et al., 2019). Cavin-4 (MURC) has been associated with dilated cardiomyopathy (Rodriguez G. et al., 2011; Szabadosova et al., 2018).

Biogenesis and stabilization of caveolae are further supported by accessory proteins, such as membrane curvature–regulating protein PACSIN2/Syndapin II, Eps-15 homology domain 2 ATPase, and receptor tyrosine kinase–like orphan receptor 1 (Lamaze et al., 2017). Both syndapin II (Koch et al., 2012; Senju and Suetsugu, 2015) and III (Seemann et al., 2017) were shown to be important in membrane shaping and stabilization of caveolae.

Apart from proteins, caveolae have a high cholesterol content, sphingomyelin, and phosphatidylserine to serve as anchoring points for cavins (Lamaze et al., 2017). The reverse of this interaction—how caveolae and their protein constituents affect lipid metabolism—is less studied (Ariotti et al., 2014; Chen et al., 2014; Golani et al., 2019).

Clustering of caveolae has been observed in different cells as “caveolar cluster-rosettes” or “multiple bulbs packed in a flower-like fashion,” and mechanisms of assembling in superstructures have been examined by computational analysis (Golani et al., 2019). The assumption has been that attractive forces originating from the energy of membrane deformation generated by the bulbs are responsible for this feature. The mechano-protective role of these structures (Szabadosova et al., 2018) putatively relays on the ability to disintegrate upon cell stretching (Golani et al., 2019). Formation of clusters of caveole is promoted by caveolar neck proteins EDH 1, 2, and 4, which partially compensate for each other to protect the cell against mechanical stress (Yeow et al., 2017).

Novel imagistic methods began to call into question some of putative characteristic features of caveolae: the specific smooth omega shape was actually an artifact of glutaraldehyde fixation (Schlormann et al., 2010), high resolution scanning EM and quick-freeze deep-etch techniques detected striations or ridges on the cytoplasmic side (Izumi et al., 1991), also detectable on flattened caveolae (Rothberg et al., 1992). A 3-D electron tomographic study showed a spiral organization of the coating (Lebbink et al., 2010), adding evidence to previous suggestions that caveolae are indeed covered by a spiky coat (Richter et al., 2008). This protein complex has been designated the caveolar coat complex, consisting of Cav-1, Cav-2 and cavins 1, 2, and 3, found in a rather strict stoichiometry of cavin-1: total caveolin—1:4. Cavins 2 and 3 were detected in a ratio of 1:2 to cavin-1 (Ludwig et al., 2013). This stoichiometry was confirmed by further studies and the coatomer organization of the caveolae was unraveled. Cavin-1 homotrimers or heterotrimers of cavins (cavin 1 to either cavin 2 or 3 in a stoichiometry of 2–3:1) are the core constituent of the coat, interacting with approximately 12 caveolin molecules (Krijnse Locker and Schmid, 2013). Using single-molecule analysis of fluorescently tagged cavins, Gambin et al. (2013) showed that in cavin hetrotrimers, expression of cavin 2 and 3 was mutually exclusive and the ratio of cavin 1: cavin 2 may vary, whereas the ratio of cavin 1: cavin 3 was 3:1. A ratio of Cav-1:cavin 1 of 3–4:1 was also confirmed. Cryoelectron tomography revealed that this caveolar coat has an inner layer composed of caveolins that assemble into a polyhedral cage, and a peripheral filamentous layer composed of cavins (Ludwig et al., 2016), responsible for the spiral aspect noticed in deep-etch studies. Using mutational analysis and cryoelectron investigations, Stoeber et al. (2016) proposed a regular dodecahedron model for the cavin coat Cav-1 oligomers associate into discs that occupy the faces of the dodecahedron. Superresolution microscopy studies further proposed a modular superstructure of caveolae, constructed on smaller scaffolds of Cav-1 oligomers, which can dimerize and oligomerize into the polyhedral caveolae coat (Khater et al., 2018, 2019a,b).

From a functional point of view, caveolae initially were considered to be endocytotic vesicles, but following early ultrastructural reports, Popescu et al. (1974) proposed a specific role in calcium signaling and smooth muscle contraction. That step opened the way to the “renaissance in thinking of caveolae as organizing centers for signal transduction” (Ostrom and Insel, 1999), a shift in scientific perception supported by identification and further study of the first caveolin (Rothberg et al., 1992). Ever since, opinions about what deserves the most attention in caveolins research have been shifting, from markers for caveolae to demonstrate co-localization of other proteins to these membrane-microdomains (Bilderback et al., 1997; Lupu et al., 1997; Scherer and Lisanti, 1997; Wu et al., 1997), to active players involved in protein–protein interactions and recruitment of other proteins, to caveolin scaffolding domains (CSDs) (Couet et al., 1997a). Caveolins were found to co-purify with different kinases and to participate in signaling events (Li et al., 1995, 1996a; Couet et al., 1997b). Still, interaction between caveolins and various proteins via caveolin-binding motifs was challenged, based on the variety of the latter, their structural role and their accesibility to interacting proteins (Byrne et al., 2012; Collins et al., 2012). Nevertheless, aberrant cell signaling is a hallmark of cancer, and caveolins were reported to be deregulated in tumor pathogenesis (Razani et al., 2000; Del Pozo and Schwartz, 2007; Quest et al., 2008). An early yet continuous trend in caveolae research has been their involvement in the physiology of muscle tissue (Gabella, 1971), leading to studies of caveolins in muscle contraction (Thyberg, 2000; Betz et al., 2001; Ohsawa et al., 2004) mechanosensing (Boyd et al., 2003; Spisni et al., 2003) and muscle disease (Galbiati et al., 2001; Tanase et al., 2009). They are now also considered dynamic membrane reservoirs, providing mechanoprotection against membrane damage upon changes in membrane tensions (Sinha et al., 2011), which leads to flattening of caveolae and lateral diffusion of Cav-1, followed by slow reconstruction (Tachikawa et al., 2017).

## Caveolae in Cell Communication

To date, functional studies have shown that caveolaedo not simply convey extracellular signals but also are actively involved in their modulation. Caveolin binding is reported to inhibit kinase activity for (i) heterotrimeric G proteins, with caveolin interacting directly with multiple G protein alpha subunits, including G(s), G(o), and G(i2) (Song et al., 1996); (ii) members of the Ras superfamily, such as H-Ras (Song et al., 1996) and RhoC (Lin et al., 2005); and (iii) Src tyrosine kinases (Li et al., 1996a), acting as negative regulators and sequestering them to the plasma membrane. Caveolins also are associated with endothelial nitric oxide synthase signaling (Bucci et al., 2000) through direct protein–protein interactions (Feron et al., 1996), exerting a suppressive effect (Razani et al., 2001). Protein–protein interaction has been reported for CSDs and other kinases, such as *PKA* (Levin et al., 2006), and co-localization with Cav-1 has been demonstrated, although without confirming a physical interaction (Stubbs et al., 2005). Caveolae also harbor key proteins involved in calcium signaling (Pani and Singh, 2009), with a functional impact on striated (Bryant et al., 2014) and smooth muscle contraction, either directly (Yang et al., 2015) or indirectly via endothelial nitric oxide synthase endothelial signaling and shear stress in endothelial cells (Yamamoto et al., 2018). In cardiac muscle, Cav-3 participates in scaffolding a molecular complex centered on calcium channels (Balijepalli et al., 2006; Harvey and Hell, 2013). In neurons, Cav-1 binds to calcium-sensing proteins and mediates photoreceptor activity (Vladimirov et al., 2018), or independently of calcium, to neurotransmitter receptors (Schmitz et al., 2010; Roh et al., 2014).

Caveolae might act as reservoirs of signaling proteins, maintained in inactive forms, until the “holders” are signaled to release them. Conformational changes in caveolins are at least in part phosphorylation-dependent (Li et al., 1996b), under the control of the very same kinases that they inactivate, acting as negative feedback signals (Chen et al., 2012).

One of the few exceptions from the caveolae inhibitory effect is insulin signaling (Yamamoto et al., 1998). Early studies have indicated that in adipocytes and pre-adipocytes, downstream insulin signaling requires intact caveolae (Parpal et al., 2001). In an embryonic kidney cell line, both Cav-1 and Cav-3 could directly stimulate insulin receptor kinase activity (Yamamoto et al., 1998). Furthermore, insulin signaling triggered relocation of Glut4 to caveolae (for review, see Cohen et al., 2003; Ishikawa et al., 2005). Recently, other caveolae-associated proteins, such as EHD2 (Moren et al., 2019) and NECC2 (Travez et al., 2018), have been identified as part of the insulin-caveolae signaling mechanism. Studies using a Cavin-1 null mouse have revealed a distinct lipodystrophic, insulin-resistant phenotype (Liu et al., 2008), which also has been subsequently documented in patients who are cavin-1 deficient (Pilch and Liu, 2011). In contrast, loss of cavin-3 does not have a significant impact on adipose tissue and glucose metabolism, as shown in cavin-3 KO mice (Liu et al., 2014a).

Of note, Cav-1 retains signaling functions in the absence of caveolae, as it continues to act as a scaffolding platform for signaling proteins. In neurons, Cav-1 was proposed to scaffold signaling components promoting neuronal survival, growth cone arborization (Head et al., 2011) and axonal growth (Wang et al., 2019). Furthermore, flattening of caveolae under membrane tension might trigger itself downstream signaling by cavin-1 release (Sinha et al., 2011), protein kinase C activation (Senju et al., 2015) or cooperation with cytoskeleton (Echarri and Del Pozo, 2015).

### Oncogenic Signaling and Caveolins

A significant body of evidence involves caveolins in oncogenesis and emerged as a consequence of caveolins’ ability to suppress cellular signaling pathways. Initial reports highlighted caveolae and caveolin downregulation in transformed cells (Koleske et al., 1995; Galbiati et al., 1998; Capozza et al., 2003), and murine Cav-1 and 2 genes were mapped to a tumor suppressor locus (Engelman et al., 1998). Since then, numerous data have been collected from cell lines involving both Cav-1 and -2 (Sagara et al., 2004), [colon carcinoma (Bender et al., 2000), human breast cancer (Lee et al., 1998), and ovarian carcinoma (Miotti et al., 2005)], or only Cav-1 (Racine et al., 1999). Further data have come from studies with KO animals (Capozza et al., 2003; Williams et al., 2004) and human tissue samples of human colon carcinoma (Bender et al., 2000), pancreatic adenocarcinoma (Tanase, 2008; Tanase et al., 2009) lung neoplasia (Kato et al., 2004), breast (Chen et al., 2004) and ovarian cancer (Wiechen et al., 2001a), and malignant mesenchymal tumors (Wiechen et al., 2001b). The role for caveolins as guardians against oncogenic transformation is supported by reports that loss of Cav-1 in tumor-associated fibroblasts drives a change in phenotype from “normal” to “fuel-supplier,” modifying the stromal environment of cancer cells into a medium favoring survival (Mercier et al., 2008; Trimmer et al., 2011).

This suppressor status has been challenged over time, however, with some limited but fairly consistent evidence of Cav-1 overexpression in various types of cancer. These findings have sometimes been in contradiction with datasets from the same types of cancer in which Cav-1 overexpression was reported (Patlolla et al., 2004) and in other cases have arisen in areas of cancer study where caveolins have otherwise gone unexamined (Ito et al., 2002; Suzuoki et al., 2002; Steffens et al., 2011). Caveolin expression seems to be increased in urogenital cancers (Kasahara et al., 2002; Fong et al., 2003; Joo et al., 2004), although not exclusively so. Some results suggest involvement of Cav-1 in resistance to cancer treatments (Sekhar et al., 2013, as reviewed in Ketteler and Klein, 2018), and in tumor spreading (reviewed in Senetta et al., 2013; Campos et al., 2019).

On the other hand, involvement of Cav-2 in tumor biology was not investigated until recently. The few data reported so far converge toward a protumorigenic role for Cav-2. In a Cav-2 KO mouse model, its loss seems to favor infiltration of tumor-associated macrophages into the tumor tissue and tumor regression (Liu et al., 2019). It also reduces metastatic potential of pancreatic cell lines (Liang et al., 2018) and its serum levels are increased in patients with pancreatic cancer, a finding associated with poor prognosis (Liang et al., 2018). Similar to Cav-1, Cav-2 involvement in tumorigenesis seems to depend on the tumor type. Investigation of Cav-2 expression in lung cancer have revealed a loss of protein during metastasis in lymph nodes, which correlated with poor prognosis (Gerstenberger et al., 2018).

The role of the cavin family as an oncogene or tumor suppressor is also controversial, depending on the cell and/or tissue type.

Most reports show the same trends for cavins as observed for Cav-1. Loss of cavin-1 is correlated with poor prognosis in colorectal cancer (Wang et al., 2017) and liposarcoma (Codenotti et al., 2017). Loss of cavin-2 also is correlated with a poor prognosis in liposarcoma (Codenotti et al., 2017) and hepatocellular carcinoma (Jing et al., 2016). In glioblastoma (Huang et al., 2018) and pancreatic adenocarcinoma, cavin-1 enhances the prognostic potency of Cav-1 (Liu et al., 2014b).

### Cell-to-Pathogen Interaction at Caveolar Sites

Caveolins were related to both antiviral and antibacterial defense. Cav-1 was proposed as a T cell–intrinsic orchestrator of TCR-mediated membrane polarity and signal specificity selectively employed by CD8 T cells to customize TCR responsiveness (Tomassian et al., 2011). Downregulation of Cav-1 apparently disperses clusters of antiviral defense–related receptors (Gabor et al., 2013), and Cav-1 KO cells display increased exosome uptake (Svensson et al., 2013). Overexpressing Cav-1 in macrophages seems to lower virion infectivity by altering cholesterol content (Lin et al., 2012) or cellular proteins required for viral replication (Simmons et al., 2012).

#### Viruses

**FIGURE 4:**
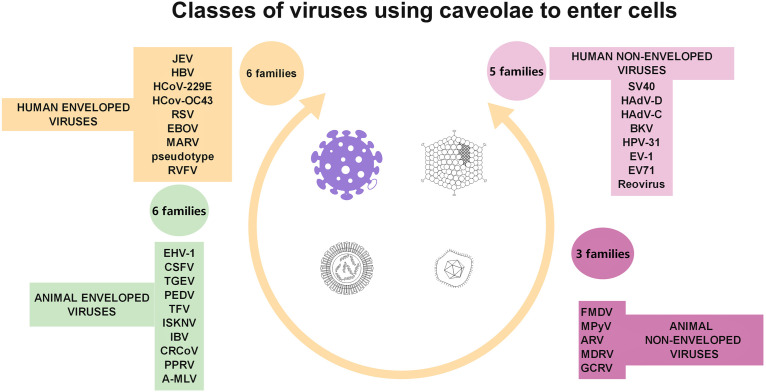
Diagrammatic representation of the four classes of viruses (taking into account virus structure and target cell type) documented to use caveolae as gates to entry cells.

Conversely, Cav-1 is a target of viral attacks, serving as a safe environment for viral replication (Yan et al., 2012), including of coronavirus strains (Nomura et al., 2004; Guo et al., 2017; Wang et al., 2020). Similar to other membrane microdomains, caveolae serve as entry gates for viruses, such as simian immunodeficiency virus (Neu et al., 2008), HCV (Shi et al., 2003), some species of adenoviruses (Leung and Brown, 2011), some herpesviridae (Hasebe et al., 2009; Kerur et al., 2010), group B of coxsackie viruses (Patel et al., 2009), foot-and-mouth disease virus (O’Donnell et al., 2008), some types of papilloma virus (Smith et al., 2007), echovirus-1 (Pietiainen et al., 2004), some coronaviruses (Nomura et al., 2004; Guo et al., 2017; Wang et al., 2020), and some types of flavivirus (Zhu et al., 2012). Viral hijacking of caveolae is summarized in Figure 4 (based on Xing et al., 2020).

A few viruses even seem to incorporate Cav-1 into new virions (Brown et al., 2002; Laliberte et al., 2006; Ravid et al., 2010). Whether caveolins are only part of the “vessel” or are being hijacked and used as “anchors” has been elucidated for some pathogens. Molecular modeling and simulation have suggested the existence of caveolin-binding sites for SARS-CoV proteins (Cai et al., 2003), although a Cav-independent mechanism also has been described (Wang et al., 2008), and for rotavirus endotoxin (Mir et al., 2007), HIV envelope proteins (Huang et al., 2007), human influenza A viruses (Sun et al., 2010), and murine leukemia viruses (Yu Z. et al., 2006).

#### Bacteria

Recent results offer growing evidence of Cav-1 acts against bacterial infections, as well (Tsai et al., 2011; Hitkova et al., 2013). It plays a protective role against fibronectin-binding pathogens, such as *Staphylococcus aureus* (Hoffmann et al., 2010) and *Pseudomonas aeruginosa* (Gadjeva et al., 2010), prevents *Neisseria gonorrhoeae* uptake (Boettcher et al., 2010), and is a critical protective modulator of sepsis (Feng et al., 2010).

On the other hand, some strains of streptococci (Rohde et al., 2003), chlamydia (Webley et al., 2004), *Salmonella* (Lim et al., 2010), *Mycobacterium, Brucella* species, FimH expressing *E. coli* (for review see Duncan et al., 2002; Medina-Kauwe, 2007), other aggressive strains of *E. coli* (Rogers et al., 2012), *Campylobacter jejuni* (Watson and Galan, 2008), *Bordetella pertussis* (Martin et al., 2015), *Leptospira interrogans* (Li et al., 2019), and *Rickettsia* (Chan et al., 2009) also use this endocytic pathway for host infectivity. In addition, caveolae are targeted by Gram-negative bacterial outer membrane vesicles, structures involved in secretion of virulence determinants, modulation of the host immune response, and contributions to biofilm formation and stability (Sharpe et al., 2011). Gonococci use Cav-1 phosphorylation and downstream signaling to switch from local to invasive infection (Faulstich et al., 2013). Bacterial entry into cells was divided in active and passive entry (Duncan et al., 2002). *Salmonella typhimurium* was identified as an example of active entry, driven by secreted proteins, while *Chlamydia trachomatis* as a typical example of passive entering germ (Duncan et al., 2002). Caveolae-mediated endocytosis was shown to be not involved in *Clostridium difficile* toxin A endocytosis (Chandrasekaran et al., 2016). This paper illustrates a thinking pattern, suggesting that clathrin-dependent and caveolae-dependent-mediated endocytosis have to be equally explored as routes for both bacteria or bacterial products. Clathrin, caveolae, macropinocytosis and secretory lysosomes were investigated as the four major points (gates) pathogens can hijack (Eto et al., 2008). Regarding mechanisms used for hijacking, data is scares as only a few articles examined specifically this subject (Eto et al., 2008; Watson and Galan, 2008; Chi et al., 2010, 2011; Lim et al., 2014; Martin et al., 2015; Chandrasekaran et al., 2016; see also Table 1).

**TABLE 1:** Summarization of pathogens using caveolae as entry gates.

**Entity type**	**Name of species**	**Experimental findings Caveolin-dependent**	**References**
Viruses	SARS coronavirus	Molecular modeling and simulation have suggested the existence of caveolin-binding sites for SARS-CoV proteins ALSO “SARS coronavirus entry into host cells through a clathrin- and caveolae-independent endocytic pathway”	Cai et al., 2003; Wang et al., 2008
	Human coronavirus	Cav-1 knockdown by RNA interference reduced the HCoV-229E infection. As mechanism, HCoV-229E binded to CD13 in DRMs, then clustered CD13 by cross-linking AND caveolin-1 dependent endocytosis was documented by confocal microscopy; the vesicle internalization process required actin cytoskeleton rearrangements.”	Nomura et al., 2004; Owczarek et al., 2018
	Hepatitis B Virus (HBV)	“HBV requires a Cav-1-mediated entry pathway to initiate productive infection in HepaRG cells; chemical inhibitors that specifically inhibit clathrin-mediated endocytosis had no effect on HBV infection.”	Macovei et al., 2010
	Human influenza A virus (H1N1)	“Cav-1 modulated influenza virus A replication presumably based on M2/Cav-1 interaction”	Sun et al., 2010
	Human immunodeficiency virus (HIV)	“HIV infection up-regulated the expression of Cav-1 and the enhanced level of Cav-1 subsequently represses virus replication by suppressing the activity of NF-κB, promoting cholesterol efflux, and blocking the fusion steps of virus infectivity.”	Mergia, 2017
	Japanese encephalitis virus (JEV)	“JEV entered human neuronal cells by caveolin-1-mediated endocytosis,. RhoA activation promoted the phosphorylation of caveolin-1, and then Rac1 activation facilitated caveolin-associated viral internalization” ALSO “JEV enters porcine kidney epithelial PK15 cells through cholesterol- and clathrin-mediated endocytosis”	Yang et al., 2013; Xu et al., 2016
	Respiratory syncytial virus (RSV)	“Co-localization of RSV antigen and caveolae was observed by confocal microscopy.” AND “RSV exploits caveolae for its assembly, and we propose that the incorporation of caveolae into the virus contributes to defining the biological properties of the RSV envelope”	Werling et al., 1999; Ludwig et al., 2017
	Rift Valley fever virus (RVFV)	“Inhibitors and RNAi specific for macropinocytosis and clathrin-mediated endocytosis had no effect on RVFV infection. In contrast, inhibitors of caveola-mediated endocytosis, and RNAi targeted to caveolin-1 and dynamin, drastically reduced RVFV infection in multiple cell lines. These results suggest that the primary mechanism of RVFV MP-12 uptake is dynamin-dependent, caveolin-1-mediated endocytosis.”	Harmon et al., 2012
	Human papillomavirus (HPV)	“HPV type 31 (HPV31) entry and initiation of early infection events require both caveolin 1 and dynamin 2 and occur independently of clathrin-mediated endocytosis”	Smith et al., 2007
	Echovirus 1	“Immunofluorescence confocal microscopy showed that EV1, alpha 2 beta 1 integrin, and caveolin-1 were internalized together in vesicular structures. Electron microscopy showed the presence of EV1 particles inside caveolae. Furthermore, infective EV1 could be isolated with anti-caveolin-1 beads 15 min p.i., confirming a close association with caveolin-1.”	Marjomaki et al., 2002
	Murine amphotropic retrovirus (A-MLV)	“…we also found colocalization of fusion-defective fluorescent A-MLV virions with caveolin-1 in NIH 3T3 cells.” CONTROVERSIAL “A-MLV is internalized not by caveolae or other pinocytic mechanisms but by macropinocytosis. A-MLV infection of mouse embryonic fibroblasts deficient for caveolin or dynamin, and NIH 3T3 cells knocked down for caveolin expression, was unaffected.”	Beer et al., 2005; Rasmussen and Vilhardt, 2015
Bacteria	Listeria monocytogenes	Cav1 Cav2 and PACSIN2 promote L. monocytogenes protrusion engulfment during spread.	Sanderlin et al., 2019
	*Edwardsiella tarda*	Entry of *E. tarda* into macrophages is clathrin- and caveolin-mediated endocytosis and cytoskeletons, and that the intracellular traffic of *E. tarda* involves endosomes and endolysosomes.”	Sui et al., 2017
	*Salmonella typhimurium*	Over-expression of Cav-1 increased Salmonellae invasion in non-senescent cells. Presence of high expression of Cav-1 in Peyer’s patch and spleen, “might be related to the increased susceptibility of elderly individuals to microbial infections” “a new model in which caveolin-1 might be involved in Salmonella entry via its interaction with SopE and Rac1, leading to enhanced membrane ruffling for phagocytosis into host cells.”	Lim et al., 2010, 2014
	*Ehrlichia chaffeensis* Anaplasma phagocytophilum	“*E. chaffeensis* and *A. phagocytophilum* utilize caveolae-mediated endocytosis for host cell entry”	Lin and Rikihisa, 2003; reviewed in Samanta et al., 2017
	*Escherichia coli*	RNA(i) reduction of cav-1 expression inhibited bacterial invasion; (iii) a signaling molecule required for E. coli invasion was located in lipid rafts and physically associated with caveolin-1; (iv) bacterial invasion was inhibited by lipid raft disrupting/usurping agents.”	Duncan et al., 2004
	*Pseudomonas aeruginosa*	“*P. aeruginosa* colonized cav1 KO mice much better compared with the wild-type controls in a model of chronic infection, indicting an important contribution of Cav-1 to innate host immunity to *P. aeruginosa* infection in the setting of both acute pneumonia and chronic infection typical of cystic fibrosis” CONTROVERSIAL “Unlike wild type mice, which succumb to pneumonia, caveolin-deficient mice are resistant to Pseudomonas”	Gadjeva et al., 2010; Zaas et al., 2009
	**Mycobacterium tuberculosis**	“… cav-1 proteins are present in great numbers in the plasma membrane of myeloid-derived suppressor cells (MDSC)”	Kotze et al., 2020
Parasites	***Trypanosoma cruzi***	“CD-1 mice infected with the Brazil strain of ***T. cruzi*** displayed reduced expression of Cav-3 and activation of ERK 66 days post infection.” **AND** “Immunofluorescence analysis demonstrated a colocalization of GM1, flotillin 1 and caveolin 1 in the ***T. cruzi*** parasitophorous vacuole of macrophages.”	Barrias et al., 2007; Adesse et al., 2010
	***Leishmania***	“…virulent L. chagasi localize in caveolae during phagocytosis by host macrophages, and cholesterol-containing macrophage membrane domains, such as caveolae, target parasites to a pathway that promotes delay of lysosome fusion and intracellular survival.”	Rodriguez et al., 2006

Some other papers were mentioning bacterial entry, but in a broader context, examining, for example, clathrin-independent mechanisms of endocytosis. It is noteworthy to observe that “nomenclature is imprecise,” criteria used to classify such transport systems relying on “cargoes, or plasma membrane markers, or carrier morphologies, or speed of the process” (for details see Ferreira and Boucrot, 2018).

Cav-1^–/–^ mice show an increased production of inflammatory cytokines, chemokines (Codrici et al., 2018), and nitric oxide and an inability to control systemic infection by *Salmonella*. The increased chemokine production in these mice leads to greater infiltration of neutrophils into granulomas but no changes in the number of granulomas present. Cav-1^–/–^ macrophages show increased inflammatory responses and increased nitric oxide production *in vitro* in response to *Salmonella* lipopolysaccharide. These results show that Cav-1 plays a key role in regulating anti-inflammatory responses in macrophages. These data collectively suggest that the increased production of toxic mediators from macrophages lacking Cav-1 is likely to be responsible for the marked susceptibility of Cav-1–deficient mice to *S. enterica serovar Typhimurium* (Medina et al., 2006).

#### Prions

PrP(C) were shown to be associated in rafts with caveolin-1 and signaling molecules, including Fyn and Src tyrosine kinases (Taylor and Hooper, 2006; Toni et al., 2006). These data open the avenue for exploring conditions in which cell are handling prion-like proteins (i.e., neurodegenerative diseases; Muradashvili et al., 2016; Puangmalai et al., 2020).

#### Parasites

Last, but not least, parasitic pathogens such as *Leishmania infantum* (*Leishmania chagasi*) (Rodriguez et al., 2006), *Plasmodium vivax* (Bracho et al., 2006), and *Trypanosoma cruzi* (Soeiro Mde et al., 1999) also use caveolae as cellular entry point. “*Leishmania* spp. include the infectious promastigote and the replicative intracellular amastigote.” It was shown that caveolae are contributing to uptake and intracellular survival of virulent promastigotes by macrophages (Rodriguez N. E. et al., 2011). Parasites of the genus *Plasmodium* induce changes within the host cell, among which, a type of “caveola-vesicle complex” (Sherling and van Ooij, 2016), defined also as “distinctive caveolae nanostructures” (Malleret et al., 2015). Chagas disease is caused by the protozoan parasite *Trypanosoma cruzi*. Cardiac injury observed during chagasic cardiomyopathy imply caveolae components (reduced expression of caveolin-3) contributing to the feature of disease (Adesse et al., 2010). Caveolae/raft-mediated endocytosis was demonstrated as the main route to AgB internalization, “a major component of Echinococcus granulosus metacestode hydatid fluid” (da Silva et al., 2018).

Of note, as presented in table 1, caveolae are not exclusive entry gates for these pathogens, who “hijack” other cellular entry pathways [for an update on bacterial manipulation of clathrin see (Latomanski and Newton, 2019), for endocytotic mechanisms used by viruses, (Slonska et al., 2016)and for parasites (Horta et al., 2020)]. Furthermore, lipids enriched in caveolae could play a part in virus entry (Ewers et al., 2010)and virus replication (Favard et al., 2019), but can be found elsewhere in the plasma membrane. This may explain partly why pathogens can enter the cells via caveolae with more or less specificity.

Current data support the idea that functions of caveolae may be hijacked by different means, in different target cells, with different consequences. Diverse viruses, prions, bacteria or parasites are capable of such hijacking. From this point of view caveolae could be considered as potential hijackable cell gates. Identification of key points during the process could make more effectively the treatment of such infectious diseases. Along with these efforts, development new drug delivery techniques could become be more likely.

### Gatekeepers of Aging

Cho and Park proposed Cav-1 as a “gatekeeper molecule” and a “major determinant of aging process” (Cho and Park, 2005). Cav-1 is involved in the regulation of many cellular processes relevant to stem cell biology, such as growth, control of mitochondrial antioxidant levels, migration, and senescence (Baker and Tuan, 2013). The observation that caveolae are decreased in senescent cells is now more than a decade old (Somara et al., 2007; Lowalekar et al., 2012, reviewed in Nguyen and Cho, 2017) and has been associated with an apparent paradox of increased cellular caveolins (Wheaton et al., 2001). *In vitro* data have confirmed that overexpression of Cav-1 induces early senescence in different cell types (Volonte et al., 2002; Cho et al., 2004; Dai et al., 2006), and Cav-1 KO animal models have a reduced lifespan (Park et al., 2003). Several reports have focused on signaling alterations in normally and induced aging cells (Castro et al., 2004; Favetta et al., 2004). A direct correlation between caveolin deficit and senescence has been reported to involve the Mdm–p53–p21 axis (Bartholomew et al., 2009). In-depth analysis of this correlation has shown that this interaction is cavin-1 dependent (Bai et al., 2011), and through inhibition of Nrf2-mediated signaling, Cav-1 links free radicals to activation of the p53/senescence pathway (Volonte et al., 2013). The same research group has argued that Cav-1 appears to play a major role in the signaling events linking oxidative stress to cellular senescence and identified an oxidant-responsive Cav-1 promoter sequence (Bartholomew and Galbiati, 2010). Furthermore, Cav-1 deficiency inhibited cardiolipin synthesis and induced mitochondrial dysfunction (Yu et al., 2017).

Different tissue types age differently in terms of caveolae and caveolins: cardiac muscle maintains the same levels of total Cav-1 and -3, but levels of Cav-1 alpha and -3 increase with aging in purified fractions of caveolae (Ratajczak et al., 2003). Other studies have reported a decrease in Cav-3 with age in ventricular myocytes (Kong et al., 2018). Aged smooth muscle has a reduced number of caveolae and reduced levels of Cav-2 and 3, but non-altered levels of Cav-1 and cavin-1 (Lowalekar et al., 2012), and aged endothelial cells have increased levels of total Cav-1 (Yoon et al., 2010). The hippocampus of aged mice shows low Cav-1 expression, and knocking it out triggers a predisposition to an early Alzheimer’s disease–like phenotype (Head et al., 2010). Total rat brain levels of Cav-1 are decreased in aged animals (Yang et al., 2018), and skin fibroblasts upregulate Cav-1 in both chronological and UV-induced skin aging (Kruglikov et al., 2019).

### Transduction of Environmental, Non-chemical Signals

Of all environmental cues perceived by cells, caveolae play an important part in translating those involved in mechanotransduction (Rizzo et al., 2003; Sinha et al., 2011; Joshi et al., 2012). This role has been comprehensively reviewed (Nassoy and Lamaze, 2012; Shihata et al., 2016), including the involvement of caveolae in signaling events, but new facets are being constantly added to this particular facet of caveolae behavior. Caveolae sensitivity to environmental physical stimuli was being uncovered as researchers began to also reveal their involvement in cellular signaling (Rizzo et al., 1998, 2003; Spisni et al., 2003), and changes in caveolar morphology under muscle stretch were reported even earlier (Gabella and Blundell, 1978). Caveolae morphology and number, as well as caveolins expression and distribution, seem to depend on shear stress (Boyd et al., 2003), gravitational force (Grenon et al., 2013), and mechanical stretch (Sinha et al., 2011). Along with disappearance of caveolae following mechanical stress in muscle cells, Cav-1 and -3 are translocated to non-caveolar membrane sites (Kawabe et al., 2004) and Cav-1 showed increased phosphorylation (Zhang et al., 2007) in a context of increased kinase activity and signaling events. Cavins are released into the cytosol, where they form a pool for subsequent caveolar reconstruction (Tillu et al., 2015).

Although Cav-3 is muscle specific, its involvement in stretch-induced cell signaling has been less studied than that of Cav-1 and may not even be mandatory for some signaling pathways (Bellott et al., 1985). Cav-3 has been recently demonstrated to regulate IL6/STAT3 mechano-signaling (and mechano-protection) (Tate et al., 1987). Both the increase in shear stress and the cessation of flow trigger a mechano-signaling cascade that leads to the generation of reactive oxygen species (Noel et al., 2013). Through their structure, composition, and mechanical properties, caveolae limit activity of mechanosensitive ion channels, which seems to require Cav-3 (Huang et al., 2013). One hypothesis is that the reservoir of Cav-1 and glycosphingolipids can be released to control mechano-signaling (Nassoy and Lamaze, 2012).

Localization of small GTPases involved in cytoskeleton rearrangement within the caveolar compartments seems to be essential for stretch-signal transduction (Kawamura et al., 2003). Regulation of RhoA, for example, drives actomyosin contractility and other mechanosensitive pathways, suggesting that caveolae could couple mechanotransduction pathways to actin-controlled changes in tension through their association with stress fibers (Echarri and Del Pozo, 2015). Unfolding of caveolae under stress, followed by activation of Src and redistribution of caveolin and glycosphingolipids, might reflect mechanisms of the cellular adaptation to mechanical stresses (Gervasio et al., 2011). The role of caveolae as mechanosensors has been extensively reviewed (Nassoy and Lamaze, 2012; Echarri and Del Pozo, 2015).

The mechanosensing of caveolae is only partially supplemented by other cellular mechanisms, as demonstrated by KO animal models. Cav-1 KO mice have impaired-flow–induced vasodilation and flow-dependent arterial remodeling, effects that are rescued by re-expression of endothelial Cav-1 (Yu J. et al., 2006). Muscle fibers from cavin-1^–/–^ mice have a prominent sarcolemmal organization, aberrant T-tubule structures, and increased sensitivity to membrane tension, effects that could be rescued by muscle-specific cavin-1 re-expression. *In vivo* imaging of live zebrafish embryos showed that loss of muscle-specific cavin-1 or expression of a dystrophy-associated Cav-3 mutant both led to sarcolemmal damage but only in response to vigorous muscle activity (Lo et al., 2015).

Another aspect of environmental communication is extracellular matrix sensing, which triggers cytoskeleton remodeling. Disruption of lipid rafts or knockdown of Cav-1 decreased cell spreading on a stiff matrix, and this effect was mediated by β1 integrin downregulation (Yeh et al., 2017). Effects of defective extracellular matrix stiffness sensing in Cav-1 loss can be rescued by constitutive activation of yes-associated protein (Moreno-Vicente et al., 2018).

### Traffic of Macromolecules and Metabolic Regulation

Caveolae are a conserved traffic pathway for a number of proteins, including albumin transfer across the endothelium in physiological (Predescu et al., 2002) and inflammatory conditions (Hu and Minshall, 2009), matrix metalloproteinase traffic (Galvez et al., 2004), endocytosis of many surface receptors, and traffic to the Golgi apparatus and endoplasmic reticulum (Le and Nabi, 2003). Involvement of caveolin in endocytosis, as well as the dynamics of caveolae between membrane-attached and scissored state are the topics of several reviews (Parton and Richards, 2003; Lajoie and Nabi, 2010; Hubert et al., 2020).

This prompted the hypothesis that caveolae are “metabolic platforms” and “gateways for the uptake of nutrients across the plasma membrane” (Örtegren et al., 2007). Caveolin-1 has been reported to regulate metabolism of lipid droplets in adipocytes (Briand et al., 2014, reviewed in Martin, 2013) and endothelial cells (Kuo et al., 2018), including the composition in peripheral phospholipids and associated proteins (Blouin et al., 2010). CAV-1 gene polymorphisms have been reported in patients with altered lipid metabolism in adult (Mora-Garcia et al., 2017, 2018) and juvenile forms (Nizam et al., 2018). Metabolic alterations are partially mediated by impaired insulin signaling (Gonzalez-Munoz et al., 2009; Perez-Verdaguer et al., 2018; Travez et al., 2018). Conversely, it was recently shown that lifestyle interventions in patients with impaired glucose regulation changed tissular and plasma cav-1 expression (Fachim et al., 2020).

To summarize, caveolae can be viewed as “hijackable gates.” They selectively filter extracellular signals, and no general rule has yet been identified for opening or closing them. The variability of response from cell type to cell type or tumor type to tumor type can be partially explained by different interaction partners. The complete picture of caveolin and cavin alterations in any given type of tumor remains elusive. Another gap that is just gaining attention is the comparative analysis of behavior between the primary tumor and metastatic sites.

## The Foreseen Trend: Caveolin Research (Not Yet) Translated Into Clinical Practice

As a consequence of cancer-related studies, Cav-1 and -2 have been investigated as tumor prognostic markers for different types of cancers, mostly carcinomas (Sloan et al., 2009; Langeberg et al., 2010; Steffens et al., 2011; Zhan et al., 2012; Lobos-Gonzalez et al., 2014). The discovery of new communication patterns between cancer and stromal cells, in which Cav-1 seems to be an important key note for both sides (Sotgia et al., 2012), prompted a new paradigm for prognosis of certain cancer types, based on evaluation of Cav-1 in stromal cells (Witkiewicz et al., 2009; Zhao et al., 2013).

In addition to cancer, caveolins are reported to be involved as gatekeepers in a wide variety of pathological processes from neurodegeneration (Head et al., 2010) to lipodystrophy (Martin et al., 2012), atherosclerosis (Pavlides et al., 2014), pulmonary fibrosis (Tourkina et al., 2008), and glaucoma (Thorleifsson et al., 2010). Genetic polymorphisms in the Cav-1 gene are associated with transplant-related renal (Moore et al., 2010) and respiratory pathologies (Kastelijn et al., 2011). Because of its tissue specificity, Cav-3 is involved in specific muscle pathologies, ranging from myodystrophies (Minetti et al., 1998) to arrhythmias (Balijepalli and Kamp, 2008).

Following the many pieces of evidence of caveolin involvement in several pathologies, attempts have been made to target them for therapeutic development in cancer (Tamaskar and Zhou, 2008), cardiovascular diseases (Sellers et al., 2012), and kidney disease (van Dokkum and Buikema, 2009). To date, only 13 clinical trials related to caveolin are listed in the U.S. National Institutes of Health database (Table 2), and only one is registered in Europe, a 2013 clinical study regarding nab-Paclitaxel treatment in HER2-negative metastatic (stage IV) breast cancer. This ongoing European study is evaluating Cav-1 expression as a secondary prognostic marker.

**TABLE 2:** Clinical trials that include caveolin-1 as target or objective.

**No.**	**Study Title**	**Conditions**	**Interventions**	**Phase**	**Primary objective**	**Secondary objectives related to Cav-1**	**Outcome measures related to Cav-1**	**NCT Number**
**1**	Caveolin-1 and Vascular Dysfunction	Hypertension insulin resistance	Drug: para-aminohippuric Acid Drug: angiotensin II Drug: norepinephrine	Phase 1			Genetic variation at the Cav-1 locus	NCT01426529
**2**	Safety, Tolerability and Pharmacokinetic Study of LTI-03 in Healthy Adult Subjects	Idiopathic pulmonary fibrosis	Drug: Cav-1 scaffolding-protein–derived peptide (LTI-03) Drug: placebo	Phase 1			Incidence of treatment-emergent adverse events	NCT04233814
**3**	Metformin Hydrochloride and Doxycycline in Treating Patients with Localized Breast or Uterine Cancer	Breast carcinoma Endometrial clear cell adenocarcinoma Endometrial serous adenocarcinoma Uterine corpus cancer Uterine corpus carcinosarcoma	Drug: metformin hydrochloride Drug: doxycycline	Phase 2	To determine if treatment with a combination of metformin and doxycycline can increase the percentage of cells that express Cav-1 in the cancer-associated fibroblasts of patients with breast, or uterine, and cervical cancers	The effect of treatment on the expression of Cav-1 in stromal cells related to the percentage of cells expressing ER and PR for breast and uterine samples and HER2 in breast cancer samples.	Primary outcome: Change in the percent of stromal cells expressing Cav-1 at an intensity of 1 + or greater assessed by immunohistochemistry Secondary outcome: Percentage of stromal cells expressing Cav-1 in relation to the percentage of cells expressing ER and PR for breast and uterine samples and HER2 in breast cancer samples.	NCT02874430
**4**	Fat Biology, Sleep Disorders, and Cardiovascular Disease	Sleep disordered breathing Cardiovascular disease			Characterization of serum/plasma levels of Cav-1, and correlate this with the presence or absence of sleep disordered breathing, obesity, and cardiovascular disease	Not mentioned		NCT01229501
**5**	Pilot Study of Anti-oxidant Supplementation With N-Acetyl Cysteine in Stage 0/I Breast Cancer	Stage 0/1 breast cancer Post biopsy Pre-surgery	Drug: IV/oral n-acetylcysteine (NAC)	Phase 1			Primary outcome: To assess the feasibility of evaluating the effect of n-acetylcysteine on tumor cell metabolism by assessing the changes in expression of Cav-1 and MCT4 in cancer-associated fibroblasts in pre- and post-therapy breast tissue treated with NAC	NCT01878695
**8**	Schedules of Nab-Paclitaxel in Metastatic Breast Cancer	Metastatic breast cancer	Drug: nab-Paclitaxel	Phase 2			To investigate the prognostic role of putative markers (SPARC and caveolin) and assess any change in the expression of SPARC and caveolin between primary and the metastatic sites	NCT01746225
**9**	Efficacy and Safety Study of ABI-007 Plus Capecitabine as First-line Chemotherapy for Advanced Gastric Cancer Patients	Gastric adenocarcinoma	Drug: nanoparticle Albumin-bound paclitaxel	Phase 2			To identify the molecular biomarkers (such as SPARK, β-Tubulin III, caveolin, etc.) by immunohistochemical and western-blotting before and during therapy, to study the biomarkers correlations with clinical outcome and toxicity	NCT01641783
**10**	Correlation Between Blood Biomarkers and Postoperative Delirium in Elective Non-Cardiac Surgery	Postoperative delirium Elective non-cardiac surgery	Diagnostic test: neuropsychological tests				Primary outcome: Serum concentration change in biomarker of blood–brain barrier disruption Cav-1 to be measured at 2 time points	NCT03915314
**11**	Radiological and Biological Tumoral and Peri-tumoral Factors in Neoadjuvant Endocrine-treated Breast Cancers	Breast cancer	Other: shear-wave elastography Drug: letrozole Procedure: breast core biopsy Other: magnetic resonance imaging	Not applicable			Secondary outcome: evaluation of Cav-1 in peritumoral tissue by immunohistochemistry	NCT02701348
**12**	A Study of Dasatinib (BMS-354825) in Patients With Advanced “Triple-negative” Breast Cancer	Breast cancer Metastasis	Drug: dasatinib	Phase 2			Secondary outcome: evaluation of Cav-1 by immunohistochemistry	NCT00371254
**13**	Neoadjuvant Pembrolizumab(Pbr)/Nab-Paclitaxel Followed by Pbr/Epirubicin/Cyclophosphamide in TNBC	Malignant neoplasm of breast	Drug: pembrolizumab Drug: nab-paclitaxel Drug: epirubicin Drug: cyclophosphamide	Phase 2			Other outcome: Cav-1 evaluation by immunohistochemistry at baseline, after treatment, and at surgery	NCT03289819

**https://clinicaltrials.gov/ct2/results?cond=&term=caveolin&cntry=&state=&city=&dist=. Accessed 10 February 2020. Cav-1, caveolin-1; HER2, human epidermal growth factor 2; ER, estrogen receptor; PR, progesterone receptor; MCT4, monocarboxylate transporter 4*.*

As a potential drug, Cav-1 is being tested in a form of a peptide-mimetic in a US randomized, double-blind, placebo-controlled trial (NCT04233814) that is starting phase 1 in 2020. The trial will assess the initial safety, tolerability, and pharmacokinetic profile of inhaled LTI-03, a Cav-1 scaffold protein–derived 7-amino acid peptide (LTI-03) in healthy participants. The results will guide any future clinical development of LTI-03 for the treatment of idiopathic pulmonary fibrosis.

Other trials are evaluating caveolin as a prognostic biomarker of therapy response in gynaecologic cancers (NCT02874430), breast cancer (NCT01878695, NCT01746225, NCT02701348 NCT00371254), and gastric adenocarcinoma (NCT01641783), and vascular response to hypertensive stimuli (NCT01426529).

No clinical trials that involve cavin are yet available.

Cell-penetrating-peptides (CPPs) are small amino acid sequences characterized by the ability to cross cellular membranes. In therapy, they can be useful for delivering bioactive molecules, such as CPP-mediated delivery of anti-tumoral proteins (Habault and Poyet, 2019).

## Conclusion

Caveolae are complex membrane microdomains of (now better) known molecular composition, with wide tissue distribution. Two classes of proteins (caveolins and cavins) cooperate to generate a gateway involved in transduction of messages from the extracellular environment. Integrity of this gate is essential for cell signaling, metabolism, antibacterial defense, mechanoreception, and aging. Mutation or loss of caveolae proteins affects human health and may generate disease phenotypes called “caveolinopathies.” Caveolae research has only recently been oriented toward translational potential, with several clinical trials to investigate their role as prognostic biomarkers in tumors. Although various cell types (prokaryotic cells, parasitic cells, tumor cells) have learned how to hijack them, we still need to learn how to exploit caveolae and their proteins for our own therapeutic purposes.

## Author Contributions

MD and EC collected data and drafted the manuscript. CT critically revised the manuscript. MG acquired EM images and prepared the Figures 1, 2. A-ME and MH performed study conception and design, and addressed all revisions. All authors contributed to the article and approved the submitted version.

## Conflict of Interest

The authors declare that the research was conducted in the absence of any commercial or financial relationships that could be construed as a potential conflict of interest.

## Abbreviations

A-MLV: Murine leukemia virus (Retroviridae)
ARV: Avian reovirus (Reoviridae)
BKV: BK virus (Polyomaviridae)
CrCov: Canine respiratory coronavirus (Coronaviridae)
CSFV: Classical swine fever virus (Flaviviridae)
EBOV: Ebolavirus (Filoviridae)
EHV: Equine herpesviruses (Herpesviridae)
EV1: Echovirus type 1 (Picornaviridae)
EV-71: Enterovirus type 71 (Picornaviridae)
FMDV: Foot-and-mouth disease virus (Picornaviridae)
GCRV: Grass carp reovirus (Reoviridae)
HadV-C: Human mastadenovirus-C (Adenoviridae)
HadV-D: Human mastadenovirus-D (Adenoviridae)
HBV: Hepatitis B virus (Hepadnaviridae)
HCoV-229E: Human coronavirus (HCoV) 229E (Coronaviridae)
HcoVO-C43: Human betacoronavirus OC43 (Coronaviridae)
HPV-31: Human papillomavirus type 31 (Papillomaviridae)
ISKNV: Infectious spleen and kidney necrosis virus (Iridoviridae)
IVB: Infectious bronchitis virus (Coronaviridae)
JEV: Japanese encephalitis virus (Flaviviridae)
MARV: Marburg virus (Filoviridae)
MDRV: Muscovy duck reovirus (Reoviridae)
MpyV: Mouse polyomavirus (Polyomaviridae)
PEDV: Porcine epidemic diarrhea virus (Coronaviridae)
PPRV: Peste des petits ruminants virus (Paramyxoviridae)
Reovirus: Reovirus (Reoviridae)
RSV: Respiratory syncytial virus (Paramixoviridae)
RVFV: Rift Valley fever virus (Phenuiviridae)
SV40: simian virus 40 (Polyomaviridae)
TGEV: transmissible gastroenteritis virus (Coronaviridae)
TVF: tiger frog virus (Iridoviridae).
